# Harnessing the yeast *Saccharomyces cerevisiae* for the production of fungal secondary metabolites

**DOI:** 10.1042/EBC20200137

**Published:** 2021-07-26

**Authors:** Guokun Wang, Douglas B. Kell, Irina Borodina

**Affiliations:** 1 The Novo Nordisk Foundation Center for Biosustainability, Technical University of Denmark, Kongens Lyngby 2800, Denmark; 2 Department of Biochemistry and Systems Biology, Institute of Systems, Molecular and Integrative Biology, University of Liverpool, Liverpool, United Kingdom

**Keywords:** fungal secondary metabolites, high-throughput screening, metabolic engineering, Saccharomyces cerevisiae, yeast

## Abstract

Fungal secondary metabolites (FSMs) represent a remarkable array of bioactive compounds, with potential applications as pharmaceuticals, nutraceuticals, and agrochemicals. However, these molecules are typically produced only in limited amounts by their native hosts. The native organisms may also be difficult to cultivate and genetically engineer, and some can produce undesirable toxic side-products. Alternatively, recombinant production of fungal bioactives can be engineered into industrial cell factories, such as aspergilli or yeasts, which are well amenable for large-scale manufacturing in submerged fermentations. In this review, we summarize the development of baker’s yeast *Saccharomyces cerevisiae* to produce compounds derived from filamentous fungi and mushrooms. These compounds mainly include polyketides, terpenoids, and amino acid derivatives. We also describe how native biosynthetic pathways can be combined or expanded to produce novel derivatives and new-to-nature compounds. We describe some new approaches for cell factory engineering, such as genome-scale engineering, biosensor-based high-throughput screening, and machine learning, and how these tools have been applied for *S. cerevisiae* strain improvement. Finally, we prospect the challenges and solutions in further development of yeast cell factories to more efficiently produce FSMs.

## Fungal secondary metabolites and their production

During long-term natural evolution, fungi, as other organisms, have developed a variety of mechanisms to gain growth advantage. Fungal secondary metabolites (FSMs), a large class of specialized small molecules, can stimulate the survival and reproduction of the host or inhibit these processes in competing organisms [1]. Bu’lock et al. [2] distinguished ‘secondary metabolites’ from ‘primary or general metabolites’ as having ‘a restricted distribution (which is almost species-specific) and no obvious function in general metabolism’. Mycotoxins (such as aflatoxin and deoxynivalenol) and antimicrobial compounds (such as penicillins, cladosporin etc.) might inhibit the growth of other microorganisms competing for limited nutrients [3]. Conversely, phytohormones and oligosaccharides could serve as plant growth enhancers and help to enable symbiotic growth [4]. Taken together, both antagonistic and symbiotic effects could support the survival and growth of FSM-producing fungi under natural conditions.

As a result of their antibacterial, antifungal, antitumor, antioxidant, and plant growth-regulating bioactivities [5–7], FSMs (as a class of natural products [8,9]) have enormous potential applications as pharmaceuticals, nutraceuticals, and agrochemicals. However, FSM production in the native host is generally low and would largely be unable to meet the demand. Historically, the production of FSMs could be improved mainly by laborious random mutagenesis and strain selection. This approach was mostly executed for antibiotics, for instance, for the improvement of penicillins production by generating a series of mutants [10]. Considering the genetic instability of the mutagenized strains, it would be more direct and effective, where possible, to improve the production by rational manipulation of the biosynthetic pathways [11]. Thanks to the development of recombinant DNA technology in the 1980s, the overexpression of short biosynthetic pathways (for example, a three-gene penicillin pathway) could be realized by the late 20^th^ century and was able to increase product titers with much greater efficiency [12,13].

**Figure 1:**
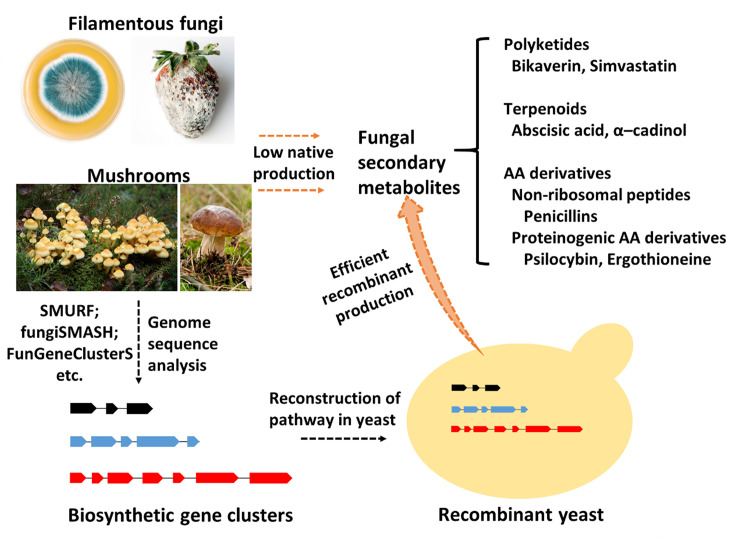
Recombinant production in yeast acts as a more efficient approach for FSMs derived from filamentous fungi and mushrooms

Although fungi do not make polycistronic operons, it has become clear, since the time when the pathway genes for penicillin were characterized, that the biosynthetic pathway genes for an FSM were often located contiguously as a biosynthetic gene cluster (BGC) in the genome. Recently, the identification of BGCs became much more straightforward due to the development of next-generation and long-read sequencing and bioinformatics tools, such as SMURF [14], AntiSMASH [15] etc. However, the validation of any BGC function is somewhat difficult using the native system due to (i) the typical non-expression (‘silence’) of the gene cluster in the native host, and (ii) the lack of genetic tools for the native host. These difficulties become even greater when it comes to strain engineering for increasing the titer of a desired product, where both the genes of the long biosynthetic pathway need to be overexpressed and the primary metabolic network is poorly characterized but likely needs rewiring. One approach to get around this is to transfer the biosynthetic pathway into a yeast host (Figure 1), for which advanced genetic tools have been well developed and where global metabolism is relatively well-studied [16–18]. Once the pathway genes are functionally expressed, the relevant FSMs are produced from the native yeast primary metabolites, precursors. Yeast chassis can be optimized via metabolic engineering to improve the supply of the required precursor molecule(s). Various other metabolic engineering strategies can be applied to further improve the production of the target molecules; these strategies will be discussed in the further sections.

## Heterologous production of FSMs in the yeast *S. cerevisiae*

### Diversity of FSMs

**Figure 2:**
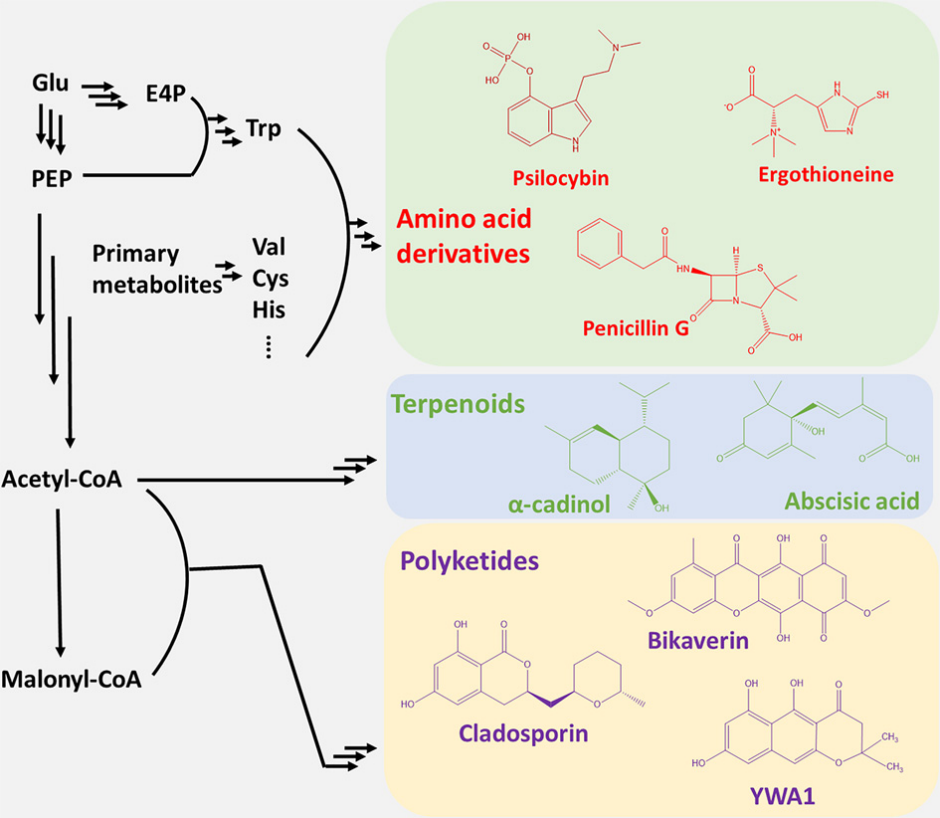
Schematic biosynthetic pathway towards typical fungi-derived AA derivatives, terpenoids, and polyketides Cys, cysteine; E4P, erythrose-4-phosphate; Glu, glucose; His, histidine; PEP, phosphoenolpyruvate; Trp, tryptophan; Val, valine.

The majority of FSMs studied to date belong to polyketides, terpenoids, and amino acid (AA) derivatives (Figure 2). Polyketide synthesis originates from acetyl-CoA/propionyl-CoA/malonyl-CoA/methylmalonyl-CoA and is driven by polyketide synthases (PKSs). Terpenoids are synthesized by terpene synthases (TSs) or terpene cyclases (TCs) from isoprene units (including geranyl and farnesyl pyrophosphates) that are also derived from acetyl-CoA. AA derivatives include non-ribosomal peptides (NRPs) and other proteinogenic and non-proteinogenic AA derivatives. NRPs are commonly formed by the condensation of proteinogenic AAs and other building blocks via multidomain NRP synthetases (NRPSs). Other proteinogenic AA derivatives are synthesized through AA modifications and condensations by a set of separate enzymes. There also exist polyketide, terpenoid, and/or NRP hybrid FSMs synthesized by multifunctional enzymes such as PKS-NRPS or PKS-TC, as well as mixed fatty acid derivatives and ribosomally derived peptides [19]. These compounds were, however, rarely investigated for recombinant production in a heterologous host and are not addressed in this review.

### Polyketides

**Figure 3:**
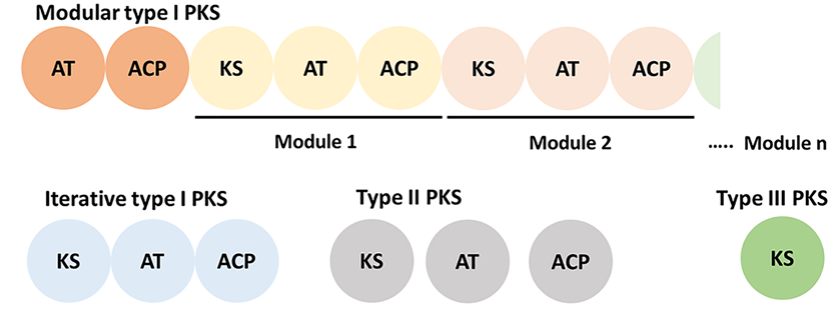
The structure of different types of PKSs The classification of PKSs is determined by the organization of domains. Functional domains/components are present either in the multidomain (type I) or dissociated (types II and III) form. Type I PKSs consist of multiple modules (modular) or a single module (iterative) where each of the active site is used once or multiple times for the assembly reactions, respectively.

Polyketide synthesis relies on the successive condensation of building blocks (CoAs) and is catalyzed by PKSs that consist of one to many functional modules. Based on the module’s organization, PKSs are roughly classified into Types I–III. Type I are large multimodule PKSs, Type II PKSs comprise multiple dissociated monofunctional proteins, and Type III PKSs are sole ketosynthase proteins (Figure 3) [20,21]. Type I PKSs are further divided into modular and iterative PKSs (Figure 3). Modular PKSs consist of multiple modules, where each active site is used only once and the molecule is assembled as it passes through the modules. Iterative PKSs consist of a single module, where the active sites are re-used multiple times [22].

**Figure 4:**
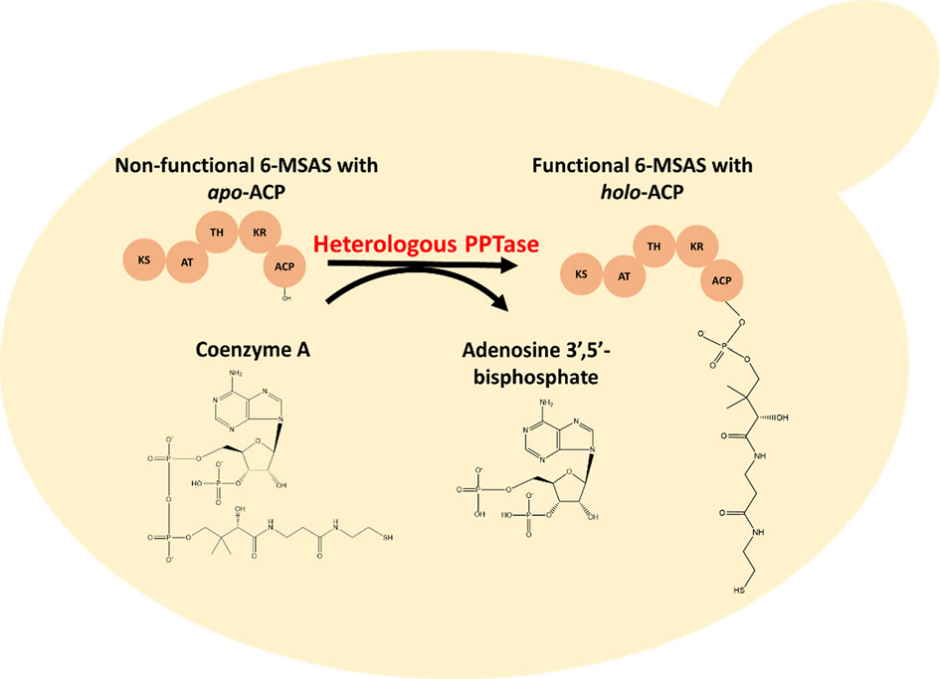
Expression of a heterologous PPTase is required for the activation of ACP domain in PKSs and NRPSs in yeast 6-MSAS is shown here as an example. In *S. cerevisiae*, there is no native PPTase that is competent for a sufficient ACP domain activation of 6-MSAS.

Depending on the complexity of PKS, the chemical structure of polyketides varies considerably in terms of chain length, the number of carbonyl groups, methylene groups, hydroxyl groups, and double bonds etc. [23]. The first recombinant polyketide produced in yeast was 6-methylsalicylic acid (6-MSA) [24], a simple model compound that could activate plant disease resistance [25]. 6-MSA is synthesized from acetyl-CoA and malonyl-CoA by 6-MSA synthase (6-MSAS), an iterative type I PKS with five domains. The domains are β-ketoacyl synthase (KS), acyltransferase (AT), thioester hydrolase (TH), ketoreductase (KR) and acyl carrier protein (ACP), where ACP, in its *apo*-ACP state, needs post-translational modification by phosphopantetheinyl transferase (PPTase) to be active (*holo*-ACP) (Figure 4). Although PPTase is present in yeast, as a module of fatty acid synthase [26], it is incompatible with and insufficient to activate heterologous PKSs, therefore, the function of PKSs requires expression of a heterologous PPTase. Kealey et al. co-expressed the genes encoding 6-MSAS from *Penicillium patulum* and PPTase from *Bacillus subtilis* (Sfp) in *Saccharomyces cerevisiae* strain InvSc1 [24]. The resulting engineered strain could produce 1.7 g/l 6-MSA in a small-scale cultivation (50 ml), while the strain expressing only the *6-MSAS* gene had no detectable 6-MSA [24]. This titer is more than two-fold higher than that achieved using the native host *P. patulum* [24]. Curiously, expressing the same gene combination in another *S. cerevisiae* strain CEN.PK resulted in only 0.05 g/l 6-MSA, while replacing PPTase with the variant from *Aspergillus nidulans* (npgA) resulted in a four-fold titer improvement, giving 0.2 g/l 6-MSA [27]. The differences between the two studies can be explained by different strain backgrounds, usage of different genetic constructs, and variations in media and cultivation conditions. A ten-fold difference in vanillin-β-glucoside production was observed between S288c and CEN.PK strains [28]. A two-fold difference in *p*-coumaric acid production was found in the same two strain backgrounds, however, once the strains have been engineered to optimize the supply of the precursor, the titers obtained in two strains were more similar [29]. When 12 different strains of the yeast *Yarrowia lipolytica* were tested for β-carotene production, the differences were up to six-fold between the lowest and the highest producing strains [30]. Besides finding efficient enzymes for PKS activation, increasing the supply of precursor, malonyl-CoA, also improved 6-MSA production. Wattanachaisaereekul et al. increased 6-MSA titer by 60% by overexpressing the *ACC1* gene encoding acetyl-CoA carboxylase and thus improving malonyl-CoA production [31].

Several polyketides that have pharmaceutical effects have been recombinantly produced in yeast. Most of these previous works were limited to functional characterization of putative PKSs using *S. cerevisiae* as expression host. Zhou et al. co-expressed two iterative polyketide synthase (IPKS) genes, *Rdc5* and *Rdc1* from *Pochonia chlamydosporia*, in *S. cerevisiae* strain BJ5464-NpgA, and achieved the production of (R)-monocillin II (approx. 15 mg/l) [32], a potential anticancer polyketide. The host strain BJ5464-NpgA is vascular protease-deficient (*pep4Δ, prb1Δ*) and is able to activate ACP due to a PPTase (npgA) expression. Using the same host, Cochrane et al. expressed PKS genes *Cla2* and *Cla3* from *Cladosporium cladosporioides* and achieved the production of cladosporin (10 mg/l) (Figure 2), a tricyclic octaketide that shows antimicrobial and antimalarial effects [33]. These studies verified the function of the PKS gene clusters *in vivo* and helped elucidate their working mechanisms.

A BGC may include not only the PKSs but also the genes encoding for tailoring enzymes, which work together to elaborate the polyketide scaffold and thus generate the final product. PKS12 from *Fusarium graminearum* catalyzes YWA1 formation. YWA1 is a heptaketide pigment. It can be further converted into rubrofusarin, a pigment with antibiotic effects, by dehydratase (aurZ) and O-methyltransferase (aurJ). The genes *aurZ* and *aurJ* are located in the same BGC as *PKS12*. By expressing the *aurZ* and *aurJ* genes in addition to *PKS12* in a *S. cerevisiae* strain carrying PPTase from *Aspergillus fumigatus*, Rugbjerg et al. achieved the recombinant production of 1.1 mg/l rubrofusarin [34]. More recently, Zhao et al. demonstrated how *S. cerevisiae* could be employed both for the whole pathway validation and engineered for an improved recombinant production of bikaverin, a red-colored tetracyclic polyketide with antibacterial and anticancer activities [35]. The authors first introduced four elements from a putative gene cluster for bikaverin synthesis, which included Type I PKS Bik1, FAD-dependent monooxygenase Bik2, O-methyltransferase Bik3, permease Bik6, and PPTase from *Fusarium fujikuroi*, as well as npgA from *A. nidulans*, into the *S. cerevisiae* [35]. With the resulting strain, the authors tested the essentiality of each element and validated the order of the biocatalytic reactions (see the detailed reaction route in Figure 4 in [35]). Furthermore, by swapping the promoter for Bik genes to the *GAL1* promoter and expressing a fused gene of *Bik2* and *Bik3*, the authors improved the bikaverin production by 273-fold to 0.2 g/l [35].

Natural statins, such as lovastatin (monacolin K) from *Aspergillus terreus* or mevastatin from *Penicillium citrinum*, are polyketides that inhibit hydroxy-methyl-glutaryl coenzyme A reductase and have cholesterol-lowering effect. Monacolin J acid (MJA) is the central intermediate compound for statin synthesis. Thus, Bond and Tang introduced the pathway genes from both *A. terreus* (PKS (LovB), thioesterase (LovG), cytochrome P450 monooxygenase (LovA), cytochrome P450 reductase (CPR), and *P. citrinum* (enoyl reductase (MlcG) into *S. cerevisiae* [36]. By optimizing the expression of the biosynthetic genes, expressing *npgA*, and deleting *PYC2, PRB1*, and *PEP4* genes, the authors improved the MJA titer to 75 mg/l [36]. Using the resulting strain, the authors further introduced an AT variant (LovD9) from *A. terreus*, and established a one-pot process for the production of simvastatin (55 mg/l), a lovastatin derivative that is still one of the top-selling statins and is on the WHO list of Essential Medicines [36].

### Terpenoids

The recombinant production of fungal terpenoids in yeast is in early development in comparison to the extensive studies on plant-derived terpenoids, such as artemisinic acid and taxadiene.

Terpenoids vary in their chain lengths (monoterpene, sesquiterpene, diterpene etc.) depending on the substrate specificity of TSs. Yap et al. introduced the sesquiterpene synthase-encoding gene GME3634 from the tiger milk mushroom *Lignosus rhinocerotis* into a *S. cerevisiae* BJ5464 strain carrying *PRB1* and *PEP4* deletions, and realized the recombinant synthesis of α-cadinol, which has selective cytotoxicity towards cancer cells [37]. Fusicocca-2,10(14)-diene (FCdiene) is a tricyclic diterpene and a precursor of anticancer drugs. Arens et al. introduced FCdiene synthase-encoding gene from *Alternaria brassicicola* UAMH 7474 (*AbFS*) into *S. cerevisiae*. They further expressed two native protein variants, hydroxy-methyl-glutaryl coenzyme A reductase isoenzyme 1 missing the N-terminal domain (tHMGR1), and a mutant of transcription factor UPC2 (UPC2-1) that regulates ergosterol metabolism [38]. The resulting strain produced more FCdiene than *Escherichia coli* and *A. nidulans* strains that carried similar genetic manipulations [38], possibly due to the higher precursor supply and/or more functional expression of catalytic enzymes in *S. cerevisiae*. Follow-up studies increased the FCdiene titer to 240 mg/l in shake flask cultivation [39,40].

Trichodermol is a sesquiterpene with potential anticancer and fungicidal effects. Its production (252 μg/l) in yeast was achieved in a *S. cerevisiae* strain expressing genes that encode for trichodiene synthase (FgTRI5) from *Fusarium graminearum* and cytochrome P450 monooxygenase from *Trichoderma harzianum* (TaTRI4 and TaTRI11), as well as overexpression of the native tHMGR1 and UPC2-1 enzymes [41].

Abscisic acid (ABA) is a phytohormone regulating plant development, senescence, and tolerance to abiotic stress. It is synthesized in plants via the cleavage of C40 carotenoids, while in fungi like *Botrytis cinerea*, it is formed by cyclization of farnesyl diphosphate (FPP) and additional modifications. Otto et al. transferred ABA biosynthetic pathway genes, viz. cytochrome P450 monooxygenase (*bcaba1 bcab2*), sesquiterpene cyclase (*bcaba3*), short-chain dehydrogenase/reductase (*bcaba4*), as well as the cytochrome P450 reductase (*bccpr1*) from *B. cinerea*, into *S. cerevisiae*. They further deleted lipid phosphate phosphatase (*LPP1*) and diacylglycerol pyrophosphate (DGPP) phosphatase (*DPP1*) genes, swapped ERG9 promoter to glucose-dependent *HXT1* promoter, and overexpressed *ERG20* and *tHMG1* genes, leading to a strain that produced 11 mg/l ABA [42].

Lycopene, responsible for the red color of tomatoes, is a tetraterpene that has potential effects in inhibiting prostate cancer [49] and cardiovascular disease [50]. Its biosynthesis involves geranylgeranyl diphosphate (GGPP) synthase (CrtE), phytoene synthase (CrtB), and phytoene desaturase (CrtI). In developing efficient *S. cerevisiae* cell factories for lycopene production, these three enzymes from various organisms (plant, red yeast, mold, and bacterium) have been tested [51–54]. Among others, CrtI from the filamentous fungus *Blakeslea trispora* (BtCrtI) was identified to contribute to the most efficient lycopene production [51,53].

### AA derivatives

Many NRPs have antibiotic activity. As with the studies of polyketides, the recombinant production of NRP in yeast was also initiated with a simple compound, δ-(l-α-aminoadipyl)-l-cysteinyl-d-valine (ACV), a penicillin precursor. Its synthesis starts with the condensation of cysteine, valine, and α-amino adipic acid. The production of ACV was achieved by reconstructing the NRP synthetase (pcbAB) from *Penicillium chrysogenum* and PPTase from either *A. nidulans, P. chrysogenum*, or *B. subtilis*, with a specific yield of 1 μg–1 mg/g dry cell weight [43]. The production of a classical antibiotic, penicillin G (benzylpenicillin), has also been realized in *S. cerevisiae*. In addition to the ACV biosynthetic pathway genes (*pcbAB* and *npgA*), the isopenicillin N synthase (*pcbC*), phenylacetyl CoA ligase (*pclA*), and acyl-coenzyme A:isopenicillin N acyltransferase (*penDE*) genes from *P. chrysogenum* were implemented. The expression levels were tuned by searching for the most productive strain within a yeast library in which genes were driven by different promoters with varying strengths [44]. The strain could produce 3 µg/l penicillin G that showed the same bioactivity as a commercial standard [44]. It is noted that the reached production level is still much lower (6 orders of magnitude) in comparison to that of optimized *P. chrysogenum* strains. The production of cyclooligomer depsipeptides—beauvericins and bassianolide, which show anticancer and antibiotic effects, was also achieved in *S. cerevisiae* [45]. Beauvericin synthetase (*BbBEAS*) and ketoisovalerate reductase (*KIVR*) genes from *Beauveria bassiana* were introduced into a BJ5464-NpgA strain and led to the production of beauvericins (105.8 ± 2.1 mg/l), while the expression of bassianolide synthetase (BbBSLS) in the BJ5464-NpgA strain resulted in a titer of bassianolide of 21.7 ± 0.1 mg/l [45].

Psilocybin is a tryptophan derivative initially identified in ‘magic’ mushrooms (*Psilocybe* spp). In the human body, psilocybin is dephosphorylated into psilocin, a molecule that causes hallucinations. Psilocybin is currently investigated in several clinical trials for treatment of cluster headaches, anxiety, and depression. Milne et al. constructed psilocybin-producing *S. cerevisiae* strain by expressing tryptophan decarboxylase (CrTdc) from *Catharanthus roseus*, cytochrome P450 monooxygenase (PcPsiH), N-methyltransferase (PcPsiM), 4-hydroxytryptamine kinase (PcPsiK), and CPR (PcCpr) from *Psilocybe cubensis* [46]. The strain produced 137.1 ± 8.3 mg/l psilocybin in small-scale cultivation [46]. Following further genetic manipulation towards an elevated metabolic flux to tryptophan (*ric1*Δ, *ARO1, ARO2, ARO4^K229L^*, and *TRP2^S65R,S76L^*), the titer was improved to 200.5 ± 6.5 mg/l. In a controlled fed-batch fermentation, the strain produced 627 ± 140 mg/l psilocybin and 580 ± 276 mg/l psilocin [46].

Ergothioneine (ERG) is a promising antioxidant nutraceutical [55], formed by methylation of histidine and a following condensation with cysteine. van der Hoek et al. reconstructed ERG biosynthesis in a *S. cerevisiae* strain by expressing various gene combinations from *Neurospora crassa, Claviceps purpurea*, and *Mycobacterium smegmatis* [47]. The strain with the two copies of *NgEgt1* and *CpEgt2* produced 598 ± 18 mg/l ERG in a fed-batch fermentation in 1-liter bioreactors [47]. Another recent study exploited the application of genes from other organisms for ERG production in *S. cerevisiae*. An engineered strain carrying *Gfegt1* and *Gfegt2* from the mushroom *Grifola frondosa* could produce up to 20.61 mg/l ERG [48].

## Re-purposing biosynthetic enzymes for non-native compound synthesis

PKS and NRPS proteins are mega-enzymes comprising many domains specific to their substrates and catalytic functions. The substrate specificity can be changed by protein engineering [56,57]. This can be carried out by library screening of enzyme variants. *S. cerevisiae* has high transformation efficiencies (up to 10^8^/μg DNA) and is well amenable for construction of libraries [58]. Many high-throughput (HTP) single-cell screening methods are available, such as fluorescence-activated cell sorting (FACS) [59], microfluidic droplet screen [60], growth coupling screen [61]. Niquille et al. tagged the target substrate and a fluorescent probe to the adenylation domain (A-domain) of NRPS displayed on yeast cell surface, and leveraged FACS to engineer the A-domain towards a target substrate. They could identify variants that favor β-amino acids as a substrate, instead of the native α-amino acids [62].

Besides AA substitution, the swapping of the functional domains or motifs also serves as an approach to altering the substrate acceptance and product spectrum [63,64]. An NRPS, BbBEAS, controls the chain length of its catalytic product through the function of the C-terminal domain. A chimeric version of BbBEAS, in which the C-terminal domain was exchanged with that of BbBSLS, could not synthesize its native product, beauvericin, but instead made a new tetrameric NRP product [65]. Expression of PKSs genes *Hpm8* and *Hpm3* from *Hypomyces subiculosus* in *S. cerevisiae* resulted in dehydrozearalenol, while the β-ketoreductase domain of Hpm8 showed stringent stereospecificity. Swapping the α4β5α5α6 motif of β-ketoreductase to the homolog of another PKS, Rdc5, changed the stereospecificity and led to the synthesis of an unnatural diastereomer of dehydrozearalenol [66].

Specific fungal biosynthetic enzymes, in comparison to the analogs from other organisms, may show higher activity and were preferentially leveraged towards the construction of an efficient *S. cerevisiae* cell factory. BtCrtI from *B. trispora* has been shown to be superior to its analogs, and its manipulation lead to the most productive *S. cerevisiae* strain for lycopene production [51,53]. Furthermore, equipping *S. cerevisiae* with the fungal enzymes could result in the biosynthesis of complex xenobiotics (prenylated β-carbolines [67]) or even new-to-nature compounds (N-acetyl-4-hydroxytryptamine [46]). As the knowledge of biosynthetic enzymes is growing, so are our opportunities to create new possibly more potent bioactive molecules.

## Advanced metabolic engineering for improving secondary metabolite production in *S. cerevisiae*

In this section, we summarize the recent development of tools and engineering strategies in *S. cerevisiae* that could be employed to improve FSMs production. To improve the performance of a cell factory, the substrate-derived carbon needs to be efficiently re-directed towards the precursor metabolites and further into the target product (Figure 2). The basic metabolic engineering strategies include up-regulation of the activity and expression of the most limiting biosynthetic enzymes, as well as deletion of enzymes competing for or degrading precursor metabolites. Such modifications can be readily carried out in *S. cerevisiae* due to convenient genome editing tools, particularly CRISPR-based. Advanced CRISPR tools in *S. cerevisiae* enable various genetic manipulations: single base mutation [68]; gene overexpression [69], activation [70], repression [70,71], and knockout [69]. CRISPR-mediated gene editing further allows multiplexing up to six genome edits in a single transformation [72]. Also methods for construction of large strain libraries have been developed, such as CRISPR-AID [73] and CHAnGE [74].

The strain library can be built by altering either the expression level [60,73] or the amino acid sequence [74] of the genes that could account for the desired phenotype, for example, an improved product titer. The variant strains with the desired phenotype can be isolated through HTP screening. Such so-called direct *in vivo* genome evolution has been aided by the rapid development of HTP screening that relies on the biosensors for important precursor metabolites (malonyl-CoA [75], tyrosine [76,77] etc.) or xenobiotic compounds [78].

Xenobiotic compounds may be toxic to *S. cerevisiae* cells and therefore retard biomass accumulation. To accumulate higher biomass to support the high production, separation of product biosynthesis from biomass accumulation, both temporally and spatially, was developed. The dynamic regulation of expression of biosynthetic genes serves as a robust temporal control approach, for instance in two-stage fermentation [79,80]. The process consists of growth and production stages, in which biosynthetic genes were muted and highly activated, respectively. This design would ensure a high biomass for product synthesis and support a high production. Spatial separation strategies include co-culture [81,82] and compartmentalization [83,84], where the toxic compounds were synthesized and metabolized by a second microorganism or in subcellular organelles, respectively. This way, toxic impacts are reduced, metabolic burdens are separated, and the benefit of reaction condition (pH, precursors, cofactors) is maximized. Besides being separated from harm, strains may also be trained to resist toxicity through adaptive laboratory evolution [85,86].

Over the growth/fermentation stage, recombinant strains may get rid of the heterologous biosynthetic pathway genes that are not essential for their growth. The resulting non-producing mutants may dominate the population as a result of the growth advantage and, therefore, reduce the overall producing capacity. An emerging trend is to use population control, via the product-activated expression of essential and selective genes; this stabilizes the producing capability of the recombinant cell population [87].

The blooming of ‘deep’ machine learning [88,89] makes it more accessible to simulate cellular processes and optimize the recombinant cells despite a limited understanding of the global metabolism. It has shown success in fine-tuning the expression of a set of pathway genes towards efficient β-carotene- [90] and tryptophan-producing [91] *S. cerevisiae* strains. This application would be broadened and speeded up by the automation that would help generate larger amounts of training data for higher prediction accuracy.

## Conclusion and perspectives

The yeast *S. cerevisiae* has been developed to become a robust host strain for the recombinant production of FSMs (Table 1). It is easy to manipulate genetically and its metabolism is well studied. Many issues hindering the functional expression of fungal biosynthetic genes in yeast, such as high GC content and accurate intron splicing, have also been readily solved by codon optimization of the mature mRNA sequence and subsequent gene synthesis. Although much progress has been made, challenges still exist in developing yeast as a universal chassis strain for FSMs.

**Table 1:** Recombinant production of FSMs in *S. cerevisiae*

Compounds	Application; function	Heterologous enzymes implemented in yeast	Manipulation of yeast native genes	Titer	Source
Polyketides					
6-MSA	Agrochemical; plant disease resistance	6-MSAS from *P. patulum*, surfactin PPTase (Sfp) from *B. subtilis*	NA	1.7 g/l	[24]
6-MSA	Agrochemical; plant disease resistance	6-MSAS from *P. patulum* and PPTase (npgA) from *A. nidulans*	NA	>200 mg/l	[27]
6-MSA	Agrochemical; plant disease resistance	6-MSAS from *P. patulum* and npgA	Overexpression of acetyl-CoA carboxylase (*ACC1*)	554 ± 26 mg/l	[31]
(R)-monocillin II	Pharmaceutical; potential anticancer effect as an inhibitor of Hsp90	IPKSs, Rdc5 and Rdc1 from *Pochonia chlamydosporia*, PPTase (npgA) from *A. nidulans*	Deletion of genes that encode for vacuolar aspartyl protease (*PEP4*) and proteinase B (*PRB1*)	15 mg/l	[32]
Cladosporin	Pharmaceutical; antimicrobial and plant-growth inhibitory activities, antimalarial effect as a result of inhibition of *Plasmodium falciparum* lysyl‐tRNA synthetase	PKSs (Cla2 and Cla3) from *Cladosporium cladosporioides*, PPTase *(npgA) from A. nidulans*	Deletion of *PEP4* and *PRB1*	10 mg/l	[33]
Rubrofusarin	Pharmaceutical and food additive; pigment with antibiotic effects on both bacteria and fungi	PKS12, dehydratase (aurZ), and O-methyltransferase (aurJ) from *Fusarium graminearum*, PPTase from *Aspergillus fumigatus*	NA	1.1 mg/l	[34]
Bikaverin	Pharmaceutical; antibacterial and anticancer activities	Type I PKS (Bik1), FAD-dependent monooxygenase (Bik2), O-methyltransferase (Bik3) and PPTase from *Fusarium fujikuroi*, npgA from *A. nidulans*	NA	202.75 mg/l	[35]
Simvastatin	Pharmaceutical	PKS (LovB), thioesterase (LovG), cytochrome P450 monooxygenase (LovA), cytochrome P450 reductase (CPR), and AT variant (LovD9) from *Aspergillus terreus*, enoyl reductase (MlcG) from *Penicillium citrinum*	Deletion of *PEP4, PRB1*, and gene for pyruvate carboxylase (*PYC2*)	55 mg/l	[36]
Terpenoids					
α-cadinol	Pharmaceutical; potential anticancer effect (selective, potent cytotoxicity in breast adenocarcinoma cells)	Sesquiterpene synthase (GME3634) from *Lignosus rhinocerotis*	Deletion of *PEP4* and *PRB1*	Detectable	[37]
FCdiene	Pharmaceutical; precursor of anticancer drug fusicoccin A	FCdiene synthase from *Alternaria brassicicola UAMH 7474* (AbFS)	Overexpression of genes that encode for hydroxy-methyl-glutaryl coenzyme A reductase isoenzyme 1 missing the N terminal domain (*tHMGR1*), variant of transcription factor UPC2 (*UPC2-1*) that regulate the ergosterol metabolism	20–240 mg/l	[38–40]
Trichodemol	Pharmaceutical and agrochemical; potential anticancer agent and fungicide	Trichodiene synthase (FgTRI5) from *Fusarium graminearum* and cytochrome P450 monooxygenase from *Trichoderma harzianum* (TaTRI4 and TaTRI11)	Overexpression of *tHMGR1* and *UPC2-1*	252 μg/l	[41]
ABA	Agrochemical; phytohormone	Cytochrome P450 monooxygenase (bcaba1 bcab2), sesquiterpene cyclase (bcaba3), short-chain dehydrogenase/reductase (bcaba4), cytochrome P450 reductase (bccpr1) from *Botrytis cinerea*	Deletion of genes that encoding for lipid phosphate phosphatase (*LPP1*) and DGPP phosphatase (*DPP1*), swapping *ERG9* promoter to glucose-dependent *HXT1* promoter, overexpression of *ERG20* and *tHMG1*	11 mg/l	[42]
AA derivatives					
NRPs					
ACV	Pharmaceutical; antibiotic precursor	ACV synthetase (pcbAB) from *P. chrysogenum*, PPTase from *A. nidulans, P. chrysogenum*, or *B. subtilis*	NA	1 μg–1 mg/g dry cell weight	[43]
Benzylpenicillin	Pharmaceutical; antibiotic	npgA from *A. nidulans*, pcbAB, isopenicillin N synthase (pcbC), phenylacetyl CoA ligase(pclA), and acyl-coenzyme A:isopenicillin N acyltransferase (penDE) from *P. chrysogenum*	NA	3 µg/l	[44]
Beauvericins	Pharmaceutical and agrochemical; antibiotic, anticancer and insecticidal effects	Beauvericin synthetase (BbBEAS) and ketoisovalerate reductase (KIVR) from *Beauveria bassiana*; PPTase (npgA) from *A. nidulans*	Deletion of *PEP4* and *PRB1*	105.8 ± 2.1 mg/l	[45]
Bassianolide	Agrochemical; insecticidal effect	Bassianolide synthetase (BbBSLS) from *Beauveria bassiana*, PPTase (npgA) from *A. nidulans*	Deletion of *PEP4* and *PRB1*	21.7 ± 0.1 mg/l	[45]
Other proteinogenic AA derivatives					
Psilocybin	Pharmaceutical; treatment of various psychological and neurological afflictions	Tryptophan decarboxylase (CrTdc) from *Catharanthus roseus*, cytochrome P450 monooxygenase (PcPsiH), N-methyltransferase (PcPsiM), 4-hydroxytryptamine kinase (PcPsiK), CPR (PcCpr) from *Psilocybe cubensis*	Deletion of transcriptional regulator RIC1 gene, overexpression of genes encoding for pentafunctional AROM protein (*ARO1*), bifunctional chorismate synthase and flavin reductase (*ARO2*), 3-deoxy-d-arabinose- heptulosonate-7-phosphate (DAHP) synthase (*ARO4^K229L^*), and anthranilate synthase (*TRP2^S65R,S76L^*)	627 ± 140 mg/l	[46]
ERG	Nutraceutical; antioxidant	ERG biosynthesis protein 1 (NcEgt1) from *Neurospora crassa*, ERG biosynthesis protein 2 (CpEgt2) from *Claviceps purpurea*	NA	598 ± 18 mg/l	[47]
ERG	Nutraceutical; antioxidant	Gfegt1 and Gfegt2 from *Grifola frondosa*	NA	20.61 mg/l	[48]

NA, not applicable.

There are still many FSMs that cannot be produced in recombinant *S. cerevisiae* strains carrying the relevant BGC (19 out of 41 in an earlier report [17]). This may be due to incorrect protein folding, modification, and trafficking. Both upgrading the native protein quality control system to be compatible with the heterologous proteins and modifying the heterologous proteins to be compatible with the recombinant system (e.g. using more suitable signal peptides) can potentially relieve the problem.

The high-level production of FSMs also requires a sufficient supply of precursor metabolites, for instance, malonyl-CoA and methylmalonyl-CoA essential for polyketides. The supply of these precursors can be improved through extensive reprogramming of the central carbon flux, bioenergetics, and redox status [92,93]. Other strategies are more direct, using non-conventional yeast strains that are superior in malonyl-CoA (such as *Y. lipolytica* [94]) and protein synthesis (such as *Pichia pastoris* [95,96]) as the ‘chassis’ strain.

The secretion of metabolites by cell factories is preferred as secretion can relieve the cellular feedback inhibition and simplify the downstream product recovery [97]. While some of the FSMs (such as ABA [42], psilocybin [46]) are mostly secreted to the extracellular medium in *S. cerevisiae*, others (such as ergothionine [47], betaxanthins [77]) are partly retained in the cell. The substrate specificity of *S. cerevisiae* transporter proteins to xenobiotic compounds is unclear, which complicates rational transporter engineering to facilitate the export. However, if an HTP screening method is available, then a library of transporter knockout yeast mutants can be created and screened for improved metabolite production [77]. Sometimes, native transporters from the metabolite-producing organisms can be expressed in yeast to facilitate the export [98].

With the rapid development of synthetic biology and metabolic engineering approaches for *S. cerevisiae* and non-conventional yeasts, we will witness emergence of novel and improved yeast-based fermentation processes for the production of FSMs.

## Summary

- FSMs have broad applications as pharmaceuticals, nutraceuticals, and agrochemicals.
- Recombinant production of FSMs in yeast can allow for an efficient manufacturing process.
- Polyketides, terpenoids, and AA derivatives have been produced recombinantly in baker’s yeast *S. cerevisiae*.
- Advanced metabolic engineering approaches relying on genome-scale engineering and HTP screening speed up the development of effective yeast cell factories.

## Abbreviations

6-MSA: 6-methylsalicylic acid
6-MSAS: 6-MSA synthase
A-domain: adenylation domain
AA: amino acid
ABA: abscisic acid
ACP: acyl carrier protein
ACV: δ-(l-α-aminoadipyl)-l-cysteinyl-d-valine
AT: acyltransferase
BGC: biosynthetic gene cluster
DGPP: diacylglycerol pyrophosphate
ERG: ergothioneine
FACS: fluorescence-activated cell sorting
FCdiene: Fusicocca-2,10(14)-diene
FPP: farnesyl diphosphate
FSM: fungal secondary metabolite
HTP: high-throughput
KS: β-ketoacyl synthase
MJA: monacolin J acid
NRP: non-ribosomal peptide
NRPS: NRP synthetase
PKS: polyketide synthase
PPTase: phosphopantetheinyl transferase
TC: terpene cyclase
TS: terpene synthase
